# Isolation, Analysis and Antimicrobial Activity of the Acidic Fractions of Mastic, Kurdica, Mutica and Cabolica Gums from Genus *Pistacia*

**DOI:** 10.5539/gjhs.v4n1p217

**Published:** 2012-01-01

**Authors:** Mohammad Sharif Sharifi, Stuart Loyd Hazell

**Affiliations:** Faculty of Medicine, University of New South Wales Sydney, NSW 2052, Australia Sydney Medical School, The University of Sydney, NSW 2006 Australia Tel: 61-421-287-461 E-Mail: m.sharifi@unsw.edu.au; Faculty of Sciences, University of Southern Queensland, Australia

**Keywords:** *Pistacia lentiscose*, Atlantica, Kurdica, Mutica, Cabolica, *Helicobacter pylori*, Anti-microbial, GEMANOVA, Fusidic acid, Steroid compounds

## Abstract

The chemical entities of Mastic, Kurdica, Mutica and Cabolica gums from genus *Pistacia* have been isolated and characterised by GC-Mass Spectrometry, High Performance Liquid Chromatography and Column Chromatography. These chemical entities were screened for anti-microbial activities against nine strains of *Helicobacter pylori* and some other Gram-negative and Gram-positive bacteria. The most bioactive components were structurally analysed. These components mimic steroid compounds, in particular, the known antibiotic Fusidic acid. Some of these chemical entities have produced promising data that could lead to the development of a novel class of antimicrobial agents that may have application in the treatment of infectious disease.

Kill kinetics have been also performed, and the produced data were evaluated by Generalized Multiplicative Analysis Of Variance (GEMANOVA) for the bactericidal and bacteriostatic activities which can be clinically significant. The isolated components were all bactericidal.

## 1. Introduction

The chemical composition of mastic gum has been studied by a number of researchers. The first attempt to characterise the chemical composition of mastic gum was made in 1904 by Tschirsh and Reutter followed by Casparis and Naef 1934 (Barton & Seaone, 1956). However, they failed to demonstrate any of those components that are known to us today. The first published account detailing elements of the chemical composition of mastic gum was by Barton and Seaone (1956). They isolated and identified three crystalline compounds from the “acidic fraction” (masticadienonic, isomasticadienonic and oleanonic acids) and one compound from the “neutral fraction” of mastic gum (tirucallol) (Barton & Seaone, 1956; Seoane, 1956). In 1973 nine esters were isolated from “acidic fraction” of the galls of *Pistacia lentiscus* (*P. lentiscus*) by chromatography after methylation with diazomethane (Monaco *et al*, 1973). They also isolated eight triterpenes from neutral fraction of the galls produced by *Aploneura lentisci* (Monaco *et al*, 1973). These data were used as a source of authentic measurements of melting point (m. p.) and optical rotations.

Isolated triterpenes from the bled resin of *Pistacia vera* have been documented to show some similarity with those that had been isolated from *P. lentiscus* (Caputo *et al*, 1978). As mastic had been used as a protective layer for artistic works including painting, there was an interest in understanding the characteristics of the gum and so it was subjected to pyrolysis gas chromatography-mass spectrometry (Chiavari *et al*, 1995) for chemical identification. Similar works have been undertaken by other researchers looking for varnishes that had been used for art and paintings (Rene de la Rie, 1989; Van der Doelen, 1998; Van der Doelen & Boon, 2000; Zumbuhl *et al*, 1998). Eight components were identified by other workers with High Performance Liquid Chromatography-Mass Spectrometry (HPLC-MS) using atmospheric pressure Chemical Ionisation (APCI) (Van der Doelen *et al*, 1998).

Much of the work that had been undertaken prior to 1987 has been summarized (Mills & White, 1989). Interestingly, they have also identified components of mastic resin together with some other resins from wrecked ships of the late Bronze Age (Mills & White, 1989). While good work has been undertaken, some attempts at characterization have not been reliable and others are perhaps trivial or irrelevant.

Thin Layer Chromatography TLC has also been used for preliminary comparisons (Hairfield & Hairfield, 1990). Some of the previously identified components of mastic identified by less reliable techniques have been confirmed using different methods. For example, Papageorgiou and associates have identified ten triterpenoid acids from acidic fraction of mastic gum by Gas Chromatography-Mass Spectrometry (GC-MS) (Papageorgiou *et al*, 1997). Also, the chemical composition of the resins extracted from insect galls found on the plant species of *Pistacia* has been analysed by a number of researchers (Caputo *et al*, 1978). Apart from a couple of papers that have been published by the same authors (Ebrahimi *et-al*, 2008; Sharifi & Hazell, 2011) on sub-species of *Pistacia* *atlantica* no other work have been published.

The identified triterpenes and triterpenoids from acidic fractions of mastic gum had structures that mimic those of steroidal compounds. Therefore action was taken to search the literature with respect to possible antimicrobial activity that triterpenes and steroids may exhibit. In China ten triterpenic acids and two steroids had been isolated from the root of *Rubus* *innominatus* from the *Rosaceae* family in which some of these components were shown to exhibit antibacterial activity (Mingkui *et al*, 2003). Steroid compounds have also been isolated from the sponge *Erylus lendenfeldi* (Geodiidae) collected in the Red Sea with demonstrated antibacterial activity against *Bacillus subtilis* and *Escherichia coli* (Al-Trabeen *et al*, 2004). They have also shown antifungal activity against *Candida albicans* (Al-Trabeen *et al*, 2004). Cholic acid also exhibits a structure similar to some of the acidic fractions of mastic gum. Some novel cationic steroid antibiotics have been synthesized by conjugating tripeptides to a triamino analog of cholic acid. These compounds have demonstrated activity against Gram-negative and Gram-positive bacteria (Bangwei *et al*, 2004).

Preliminary analysis of extracts from *Bryophyllum Pinnatum* (Lam) Oken has shown the presence of steroids, flavanoids, saponins, tannins, glycosides and acids. Such extracts has shown antibacterial activity against *Escherichia coli* and *Staphylococcus aureus* (Akpuaka *et al*, 2003).

Two novel steroidal phenols have been synthesized and screened against strains of multiresistant *Staphylococcus aureus*, a vancomycin resistant *Enterococcus faecalis* and fast growing mycobacteria (Lange *et al*, 2004). Their antibacterial activity was dependant on the length of alkyl chain (Lange *et al*, 2004). Similarly, stigmasterol and β-stigmasterol glycoside were isolated and identified by 2D NMR; these compounds demonstrated significant antimicrobial activity (Nacef *et al*, 2003).

**Figure 1:**
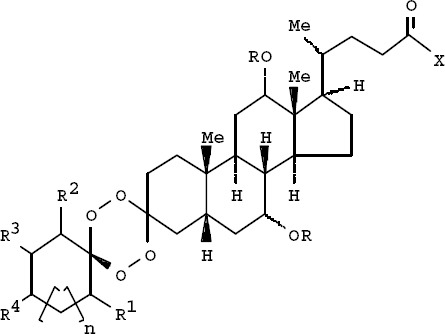
Tetraoxane derivative of steroid

A tetraoxane derivative of steroid: I [R = H, ethanoyl, propanoyl, benzoyl; R1 = H, Me, Et, isopropyl; R2 = H, Me, Et; R3 = H, Me, Et; R4 = H, Me, Et, tert-Bu, aryl, ester, etc.; X = alkoxy, amino, N-alkylamino, N-arylamino; n = 0-3], and all other possible stereoisomers has demonstrated high antimicrobial activity against the malarial parasite *Plasmodium falciparum* chloroquine-susceptible strain D6, and the chloroquine-resistant strain W2 respectively (Solaja *et al*, 2003) (Figure. 1).

The chemical compositions of gum extracted from the *atlantica* species and its sub-species are not known but are expected to be similar to that of mastic. The acidic fractions of the sub-species of *atlantica*, particularly *kurdica* have been demonstrated to contain compounds exhibiting anti-microbial activity; (Sharifi, 2006) therefore work was undertaken to identify the compositions of extracted fractions and compare the spectra with the published spectra in literature. In this study, the analysis of acidic fractions of mastic gum as a reference and kurdica, mutica and cabolica gums are reported. The identification of the GC-MS peaks was performed by published mass spectra and retention characteristics of mastic gum (Papageorgiou *et al*, 1997). The retention time and characteristics of mastic gum that was obtained by GC-MS was first verified with published data, and then the verified obtained data was used as a criterion for identification of sub-species of *atlantica*.

The acidic fractions of mastic (Sigma Aldridge), kurdica, mutica and cabolica gum (Kurdistan Saghez Sazi and Surij from Iran) were analysed using GC-MS (Sharifi, 2006). Thirteen triterpenoid acids were identified by retention characteristics as their methyl esters (i.e., Moronic acid, Oleanonic acid, Ursonic acid, Oleanolic acid, Isomasticadienonic acid, 3-epi-isomasticadienolic acid, Masticadienonic acid, Dihydromasticadienonic acid, 3-O-acetyl-3epi(iso)masticadienolic acid, Masticadienolic acid, Dihydromasticadienolic acid, 3-acetoxy-3-epiisomasticadienolic acid, 3-acetoxy-3-epimasticadienolic acid) in mastic gum. Their structures were identified by analysis of their spectral data, optical rotation and melting point and also by comparing with authentic reported data and co-injection with authentic samples (Chiavari *et al*, 1995; Papageorgiou *et al*, 1997; Van der Doelen & Boon, 2000).

This data was used to identify the chemical composition of kurdica, mutica and cabolica gums and to compare the composition by reference to their biological activity. The objectives of the work outlined in this study were as follows:

- To identify the individual components of mastic gum in relation to their antimicrobial activities.
- To use mastic gum data (GC-MS) for identification of kurdica, mutica and cabolica gums components.
- To screen for compounds in gum extracts that have antimicrobial activity seeking to identify and isolate specific compounds with substantial antimicrobial activity.
- To investigate any differences between the active components identified within the fractions.
- To perform kill kinetics on individual chemical isolates seeking to identify bactericidal or bacteriostatic activities.
- To identify any common structure/s of the active component/s.

## 2. Experimental

### 2.1 GC-MS Analysis of Acidic Fractions

The acidic fractions of the gums were extracted (Sharifi & Hazell, 2009). These fractions were dissolved in acetonitrile 11mL. This solution was then methylated. The methylated solution was evaporated under vacuum and solids were analysed by GC-MS (Sharifi, 2006). The methylated acidic fractions of kurdica, mutica, cabolica and mastic gum were analysed by GC-MS in split mode (20:1) 0.5 µm injection volume in a Shimadzu QP-5000 GC-MS System with a 30m BP-5 fused silica capillary column of 0.25 mm I.D. and 0.11µm film thickness as described previously (Sharifi, 2006). High Resolution Mass Spectrometry used to determine the molecular formula. The obtained spectrum of mastic gum was used in order to obtain a retention time for the ten previously identified methylated triterpenoid.

### 2.2 Column Chromatography

A vertical glass column was used to separate and collect the most active fractions of the gum (Sharifi, 2006). In this technique, the mixture to be analysed is placed on the top of the column and flows down through the column (by either gravity or external pressure). This process of fractionation was performed parallel to GC-MS for further validations and also to collect the fractions for antimicrobial assay in the existing form whereas in GC-MS these fractions were methylated. While the intention was to repeat the method reported in the literature (Barton & Seaone, 1956), allowing the comparison with authentic data, some modification in the mobile phase was necessary to optimize the separation. This optimization was obtained by TLC (Sharifi, 2006). Purity of the fractions was tested by Chemical Ionisation (CI) Mass Spectrometry, followed by identification by EI Mass- Spectrometry. These spectra were then correlated with authentic spectra in the literature (Chiavari *et al*, 1995; Monaco *et al*, 1973; Papageorgiou *et al*, 1997; Sharifi, 2006; Van der Doelen & Boon, 2000).

The impure sub-fractions were discarded and the pure components were then crystallized from ether/benzene or methanol where applicable. Rotations were determined in CHCL_3_, at the concentration of 0.2%, UV absorptions were taken by Varian UV spectrophotometer equipped with Carry 50 software. The molecular weight, m/z fragments, peak intensities and [α]_D_ are tabulated in Table 1, Table 1.1 and Table 1.2. The pure components were then kept for further analysis and screening for any antimicrobial activities against the strains of *H. pylori* and Gram-positive and Gram-negative.

**Table 1:** Chemical composition of acidic fractions of mastic, kurdica, cabolica and mutica gum

No.	I.Time -F.Time	Mastic %	Kurdica %	Cabolica %	Mutica %	Common name/Systematic name	mp	[α]_D__25º__CHCl3_	M	m/z characteristic ions of methylated compounds.EI (70ev) (int. %)/Under APCI
1	30.017-30.550									
2	34.267-34.6		0.71	0.39	0.53					
3	36.517-36.7		0.85		0.23					
4	37.983-38.350	11.22	11.97	7.38	8.20	Methyl moronate 3-oxo-olean-18-en-28-oic Fig 5-2 methyl ester	218-220º	+60º	468	468(61), 249(55), 189(100)
5	38.583-38.850	7.87	4.93	4.80	5.2	Methyl oleanonate 3-oxo-olean-12-en-28-oic. Fig 5-3 methyl ester	180-182º	+75º	468	468(25), 262(53) 203(100)
6	38.850-38.967		0.76		0.31	Ursonic acid methyl ester 3-oxo-urs-12-en-28-al)	174-176º		468	438(16), 232(21), 203(100)
7	38.967-39.217		2.92		2.51					
8	39.217-39.517	1.38	0.81		1.10				468	468((25), 262(53), 203(100)
9	40.200-40.733		4.46						438	438(16), 232(21), 203(100)
10	40.733-41.100	0.53	3.39	1.02	2.80	Methyloleanolate 3β-hydroxy-olean-12-en-28-oic. Fig 5-5 methyl ester	196-198º	+85º	470	470(16), 410(14), 262(70), 203(100)
11	41.100-41.517	30.74	14.79	13.16	20.50	Methyl isomasticadienonate 3-oxo-13α, 14β, 17βH,20αH-lanosla-8, 24-dien-26-oic. Fig 5-6 methyl ester	110-112º	+37º	468	468(31), 453(100), 421(21)

**Table 1.1:** Chemical composition of acidic fractions of mastic, kurdica, cabolica and mutica gum

12	41.517-41.900	0.87	1.16	0.43	1.10	Methyl 3-epi-isomasticadienolate3α-hydroxy-13α,14β,17βH, 20αH-lanosta-8,24-dien-26-oic Fig6-7 methyl ester	140-142º	+12º	470	437(100), 121(52) 95(75)
13	42.433-42.650		0.62		1.20					
14	42.233-42.433		0.58							
15	42.433-42.65		0.62							
16	43.050-43.700	40.13	20.06	21.11	32.90	Methyl masticadienonate 3-oxo-13α,14β,17βH,20αH-lanosta-7,24-dien-26-oic Fig-6.8methyl ester	123-124º	-71º	468	468(30), 453(100), 421(21)
17	43.700-43.917		0.61							
18	43.917-44.133		0.60			Methyl Dihydromasticadienonate 3-oxo-13α,14β,17βH,20αH-lanosta-7,en-26-oic methyl ester	90-92º	-75°	470	455 (50), 423 (100)
19	44.133-44.667		9.00			Methyl 3-O-acetyl-3epi-iso-masticadienolate (3α-acetoxy-13α, 14β*,* 17 βH,20αH-Ianosta 8,24-dien-26-oic acid Or 3α-acetoxy-3α,14β17 βH,20αH-Ianosta-7,24--dien-26-oic acid	85-87º	-2º	512	512(26), 497(29), 437(100)

**Table 1.2:** Chemical composition of acidic fractions of mastic, kurdica, cabolica and mutica gum

20	44.667-44.950	0.79	0.62	3.45	0.71	Methyl masticadienolate 3βhydroxy13α,14β,17βH,20αH-lanosta-7,24-dien-26-oic Fig 6-9 methyl ester	121-122º	-44º	470	455 (60), 437(100), 121(30), 95(50)
21	45.200-45.75		1.24							
22	45.817-46.283		2.29							
23	46.483-46.817		1.06			Methyl Dihydromasticadienolate 3β-H-hydroxyl 3α, 14β,17βH, 20αH-lanosta-7-en-26-oic methyl ester	115-116º	-44º	472	457 (10), 454 (80) 439 (100), 301 (10) 257 (15)
24	46.817-47.283	2.76	10.14	2.74		Methyl 3-acetoxy-3-epiisomasticadienolate 3α-acetoxy13α,14β,17βH,20αH-lanosta-8,24-dien-26-oic methyl ester Fig 6-10	118-122°	+22	512	512(21), 497(26), 437(100)
25	47.550-48.050	2.94	4.03	2.65		Methyl 3-acetoxy-3-epimasticadienolate 3α-acetoxy-13α, 14β, 17βH, 20αH-lanosta-7,24-dien-26-oic. Fig 6-11 methyl ester	100-102º	-45º	512	497(20), 437(100), 189(19), 127(25), 95(34)

### 2.3 High Performance Liquid Chromatography

High Performance Liquid Chromatography (HPLC) is a method of analysis that is not limited by the volatility or stability of the sample compound. HPLC is used to separate, identify, purify and quantify various compounds. Atmospheric Pressure Chemical Ionisation-Mass Spectrometry (APCI-MS) method was used to avoid any changes in chemical structure of triterpenoids components of the acidic fractions (Van der Doelen *et al*, 1998) and also to validate the identification made as a result of data obtained by GC-Mass and collected following column chromatography (Sharifi, 2006). The identified collected fractions were kept for antimicrobial screening against the strains of *H. pylori* and Gram-positive and Gram-negative bacteria, and also to investigate the mode of the action that will be reported in a separate paper.

## 3. Antimicrobial Activity of the Isolated Components of the Acidic Fractions

### 3.1 Minimum Inhibitory Concentration (MIC)

The MIC and MBC values were determined for all the sub-fractions of acidic (Sharifi & Hazell, 2009) and all chemical entities that are listed in Table 1, 1.1 and 1.2 against 9 strains of *H. pylori* table 2 and all other Gram-positive and Gram-negative bacteria listed in table 3 and 4 using the broth micro-dilution method (Sharifi & Hazell, 2009).

**Table 2:** The MIC values of the isolated components of the acidic fractions of the gums against the strains of *H. pylori* (µg/mL)

Sub-fractions of acidic fractions of the gums	*H. pylori* 26695	*H. pylori* J99	*H. pylori* RSB6	*H. pylori* P10	*H. pylori* SS1	*H. pylori* SS2000	*H. pylori* N6	*H. pylori* NCTC 11637	*H. pylori* RU1
Moronic acid	10	15	20	10	5	10	10	10	10
Oleanonic acid	10	10	10	10	5	10	10	10	10
Ursonic acid	50	100	50	50	50	100	50	50	50
Oleanolic acid	25	20	20	25	25	25	20	25	25
Isomasticadienonic acid	5	5	5	5	5	5	5	5	5
3-epi-isomasticadienolic acid	10	5	5	5	5	10	5	5	5
Masticadienonic acid	5	5	5	10	5	5	5	5	5
Dihydromasticadienonic acid	1	1	1	1	1	1	1	1	1
3-O-acetyl-3epi(iso)masticadienolic acid	0.01	0.01	0.01	0.01	0.01	0.01	0.01	0.01	0.01
Masticadienolic acid	5	5	5	5	5	5	5	5	5
Dihydromasticadienolic acid	1	0.5	0.5	1	0.5	1	1	1	1
3-acetoxy-3-epiisomasticadienolic acid	0.1	0.5	0.1	0.1	0.1	0.5	0.5	0.5	0.5
3-acetoxy-3-epimasticadienolic acid	0.5	0.5	0.1	0.1	0.1	0.1	0.1	0.1	0.1

**Table 3:** The MIC values of the isolated components of the acidic fractions of the gums against the Gram-negative bacteria (µg/mL)

Sub-fractions of acidic fractions ‘a’ and ‘b’ of gums	*Escherichia coli*	*Salmonella typhimurium*	*Serratia marscens*	*Pseudomonas aeruginosa*	*Alcaligenes faecalis*	*Enterobacter aerogenes*	*Pseudomonas fluorescens*	*Proteus vulgaris*	*Porphyromonas gingivalis*
Moronic acid	20	20	20	20	20	20	10	10	10
Oleanonic acid	20	20	20	20	20	10	10	10	10
Ursonic acid	50	50	50	50	50	50	50	50	50
Oleanolic acid	25	25	25	20	25	25	25	25	25
Isomasticadienonic acid	5	5	5	5	5	5	5	5	5
3-epi-isomasticadienolic acid	1	1	1	1	1	1	1	1	1
Masticadienonic acid	5	5	5	5	5	5	5	5	5
Dihydromasticadienonic acid	1	1	1	1	1	1	1	1	5
3-O-acetyl-3epi(iso) masticadienolic acid	0.01	0.02	0.01	0.05	0.01	0.01	0.01	0.01	0.01
Masticadienolic acid	2	2	2	2	2	2	2	2	2
Dihydromasticadienolic acid	1	1	1	1	1	1	1	1	1
3-acetoxy-3-epiiso-masticadienolic acid	1	1	1	1	1	1	1	1	1
3-acetoxy-3-epimasticadienolic acid	1	1	1	1	1	1	1	1	1

**Table 4:** The MIC values of the isolated components of the acidic fractions of the gums against the Gram-positive bacteria (µg/mL)

Sub-fractions of acidic fractions ‘a’ and ‘b’ of gums	*Bacillus cereus*	*Staphylococcus aureus*	*Streptococcus faecalis*	*Staphylococcus epidermidis*	*Bacillus subtilis*	*Corynebacterium sp.*
Moronic acid	50	50	75	50	100	50
Oleanonic acid	50	50	50	50	50	50
Ursonic acid	100	100	100	100	100	100
Oleanolic acid	20	20	20	25	20	20
Isomasticadienonic acid	5	5	10	5	5	5
3-epi-isomasticadienolic acid	5	5	5	5	5	5
Masticadienonic acid	5	5	5	5	5	5
Dihydromasticadienonic acid	2	5	2	5	2	2
3-O-acetyl-3epi(iso)masticadienolic acid	0.01	0.05	0.01	0.02	0.02	0.01
Masticadienolic acid	5	10	5	5	10	5
Dihydromasticadienolic acid	2	2	2	2	2	2
3-acetoxy-3-epiisomasticadienolic acid	2	2	5	2	2	2
3-acetoxy-3-epimasticadienolic acid	2	5	2	2	2	2

### 3.2 Time-kill kinetic

The 26695 strain of *H. pylori, Escherichia coli type 1 and Staphylococcus aureus* were chosen for time-kill kinetic experiments with static liquid cultures. The cultures were allowed to grow to stationary phase and that was determined by taking the Optical Density 600 (OD600) of the cultures (Ebrahimi *et-al*, 2008; Sharifi & Hazell).

The data collected from kill kinetics with MIC and 5X MIC for individual components were recorded on Microsoft Excel and analysed using Microsoft Excel, Sigma plot and MATLAB. As a large number of data was produced from the complex interactions, the classic ANOVA offered limited interpretability. Therefore, Generalized Multiplicative Analysis of Variance (GEMANOVA) method was proposed to tackle this problem in data generated mainly by these complex interactions (Ebrahimi *et-al* 2008). This method was the first application of GEMANOVA to model the data from the field of microbiology and the first GEMANOVA model in which more than two multi-way terms are used and interpreted (Ebrahimi *et-al* 2008).

### 3.3 Minimum Inhibitory Concentration

The MIC results have been tabulated in Tables 2-4 for all the components of acidic fractions of the mastic, kurdica, mutica and cabolica gums, against the strains of *H*. *pylori* Table 2 and all other Gram-positive and Gram-negative bacteria listed in Table 3-4

The MIC values for the components listed in Table 1, 1.1 and 1.2 ranged from 0.1-50μg/mL against the strains of *H. pylori* and all other Gram-negative bacteria (Table 2 and 3) and ranged from 2-100μg/mL against Gram-positive bacteria (Table 4).

### 3.4 Kill Kinetics

The rate of killing for all the components was almost constant. Statistically significant results were determined by a P value of less than or equal to 0.05 (Ebrahimi *et-al* 2008) in their respective MIC and 5MIC.

## 4. Results and Discussion

The chemical characteristic of the specific components of mastic gum, galls from *P. lentiscus* and *P. vera* are well known. Some of these characteristics were used as authentic data (Monaco *et al*, 1973).

The characteristics of isolated components of acidic fractions of mastic, kurdica, mutica and cabolica gums such as molecular weight, GC-MS data, melting point and optical rotations listed in Table 1, 1.1 and 1.2 correlate with literature (Barton & Seaone, 1956; Papageorgiou *et al*, 1997; Seoane, 1956).

When this study began in January 2000, no published data were available on antimicrobial activity of the mastic gum and other related gums and their fractions. The first abstract from this work was published on June 2001 (Sharifi *et al*, 2001). Soon after that abstract was published, a short paper was published reporting on the antimicrobial activity of whole mastic gum (not defined fractions) against *H. pylori* (Marone *et al*, 2001). In 2003 a patent was published reporting the antimicrobial activity of the mastic as a whole together with a mixture of some of the fractions, that is not pure chemical entities (Fotinos *et al*, 2003).

Important features of the chemical entities that have been identified and isolated in this study are their antimicrobial activity. Most of the chemical entities above have not been tested for antibacterial activity previously, particularly with respect to *H. pylori*. Thus it was important to further characterise these compounds with respect to their capacity to inhibit or kill bacteria.

The antimicrobial screening of these chemical entities led to fundamentally new information that went beyond *H. pylori*, expanding the original parameters of the study. Such was the extent of these findings that a new class of antibiotics may have emerged and their structure have been characterised. The mechanism of their action and structural related activities will be discussed in a separate paper. Furthermore, the potential to enhance the antimicrobial activity of antibiotics has been incremented and as a result the ability to design a new class of antibiotics has become possible.

The MIC values for moronic acid ranged from 5-20 μg/mL. *H. pylori* strain SS1 was more sensitive with MIC 5 μg/mL (Table 2). MIC values ranged for all other Gram-negative bacteria tested from 10-20 μg/mL (Table 3) and for Gram-positive bacteria ranged from 50-100 μg/mL (Table 4). Antimicrobial activity of moronic acid isolated from *Ozoroa mucronata* has been previously reported in 1979 (Hostettmann-Kaldas & Nakanishi, 1979). However, antiviral activity of this substance is well known and it is reported to be active against Herpes (Kurokawa *et al*, 1999). Purified Moronic acid from *Rhus javanica* has shown significant anti-HSV activity *in vitro* and *in vivo* with therapeutic index of (10.3–16.3). The effective concentrations for 50% plaque reduction of moronic acid for wild type HSV type 1 (HSV-1) was 3.9 mg/mL (Kurokawa *et al*, 1999). Moronic acid has also been isolated from *Myrceugenia euosma* and shown significant anti-HIV activity with therapeutic index of over 186 (Singh *et al*, 2005). This substance and its derivatives were classed “Structure I” for structural analyses (Table 5).

**Table 5:** Chemical structure of isolated components of the gums

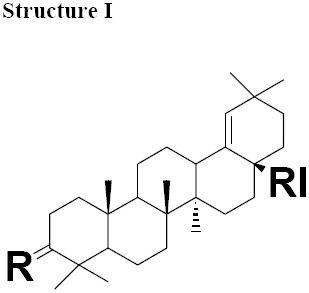	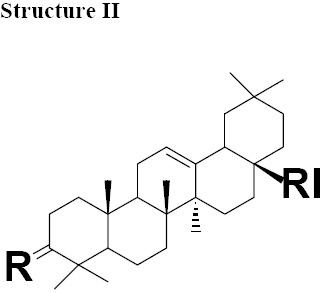	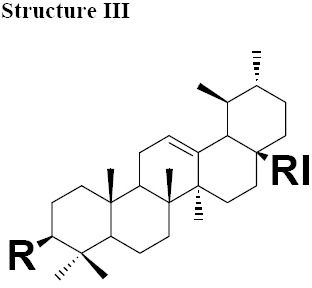
a: R= O RI= COOMe Moronate b: R= O RI= COOH Moronic acid c: R= O RI=CHO Moronic aldehyde	a: R = O, RI = COOMe Oleanonate b: R = O, RI = COOH Oleanonic acid c: R = H, β-OH, RI = COOMe Oleanolate d: R =O RI = CHO Oleanolic aldehyde	a: R = H, α-OH RI = CHO Ursonic aldehyde

The MIC values for oleanonic acid against the nine strains of *H. pylori* ranged from 5-10 μg/mL. Oleanolic acid and ursonic acid were less active against the 9 strains of *H. pylori* with MIC values ranged from 25-100 μg/mL (Table 2), for all other Gram-negative bacteria the MIC was 50 μg/mL and for all other Gram-positive bacteria the MIC was 100 μg/mL (Table 3 and 4). Antimicrobial activity of oleanonic acid and its derivatives have not been previously reported. Oleanonic and ursonic structures and their derivatives were classed “Structure II and Structure III” respectively (Table 5). Lanosta base skeletons were classed Structure IV, V and VI table 6.

**Table 6:** Chemical structure of isolated components of the gums

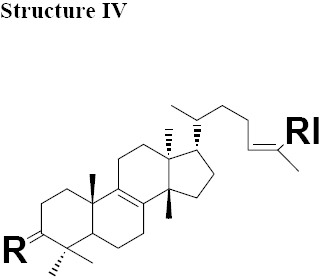	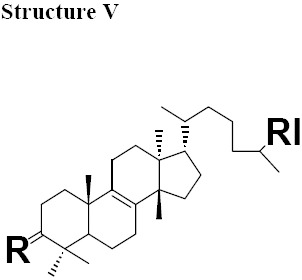	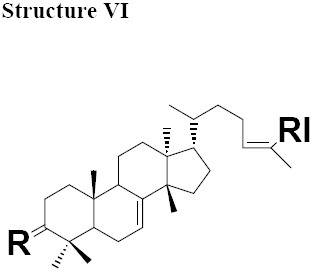
a: R = O, RI = COOMe Masticadienonate, b: R = O RI = COOH, Masticadienonic acid c: R =H, β-OH, RI = COOMe Masticadienolate, d: R =H, β-OH RI = COOH, Masticadienolic acid e: R = α-CH3COO, RI = COOMe 3α-acetoxy-3-epimasticadienolate f: R = α-CH3COO β-OH, RI = COOMe 3-α-acetoxy-masticadienonate g: R = α-CH3COO, RI = COOH 3-α-acetoxy-masticadienonic acid h: R = α-CH3COO, RI = COOMe 3-α-acetoxy-3-epimasticadienolate i: R = α-CH3COO β-OH RI = COOH 3-α-acetoxy-3-epimasticadienolic acid	a: R = O RI = COOMe Dihydromasticadienonate b: R = O RI = COOH Dihydromasticadienonic acid c: R =H, β-OH RI = COOMe Dihydromasticadienolate d: R =H, β-OH RI = COOH Dihydromasticadienolic acid	a: R = O, RI = COOMe, Isomasticadienonate b: R = O, RI = COOH Isomasticadienonic acid c: R =H, α-OH, RI=COOMe 3-epi-isomasticadienolate d: R =H, α-OH, RI = COOH 3-epi-isomasticadienolicacid e: R = α-CH3COO, RI = COOMe 3-α-acetoxy-masticadienonate f: R = α-CH3COO, RI = COOH 3-α-acetoxy-isomasticadienonic acid g: R = α-CH3COO β-OH, RI = COOH 3-acetoxy-3-epi-isomasticadienolate h: R = α-CH3COO β-OH, RI = COOH 3-acetoxy-3-epi-isomasticadienolic acid, i: R =H; α-OAc, RI = COOMe 3-O-acetyl-3epi-iso-masticadienolate j: R =H; α-OAc, RI = COOH 3-O-acetyl-3epi-iso-masticadienolic acid

MIC values for masticadienonic acid, isomasticadienonic acid and masticadienolic acid against 9 strains of *H. pylori* was 5μg/mL. Testing masticadienonic acid against the strain P10 of *H. pylori* yielded an MIC of 10 μg/mL (Table 2). Masticadienonic acid and isomasticadienonic acid had MIC values of 5 μg/mL and masticadienolic acid, 2 μg/mL against all other Gram-negative bacteria (Table 3). Their MIC values against Gram-positive bacteria ranged from 5-10μg/mL; (Table 4) these three compounds are designated structures IVb, VIb and IVd respectively (Table 6).

MIC values for dihydromasticadienonic acid against all the strains of *H. pylori* and all other Gram-positive and Gram-negative bacteria ranged from 1 to 5μg/mL. The MIC values of dihydromasticadienolic acid exhibited a tight cluster ranging from 0.5-2μg/mL. This compound is designated structure Va (Table 6).

MIC values for 3-epi-isomasticadienolic acid against *H. pylori* strains ranged 5-10μg/mL. This MIC was higher than that observed for all other Gram-negative bacteria at 1μg/mL and was similar to that observed in Gram-positive bacteria 5μg/mL (Table 2-4). No particular antimicrobial pattern with respect to Gram-positive and Gram-negative bacteria was identified. This compound exhibited broad spectrum activity with an MIC ranged of 1-10 μg/mL and is designated structure VId (Table 6).

MIC values for 3-acetoxy-3-epiisomasticdienolic acid and 3-acetoxy-3-epimasticdienolic acid ranged from 0.1-0.5μg/mL against *H. pylori*, 1μg/mL against all other Gram-negative bacteria tested with the exception of *E. coli* type 1 which had an MIC of 5μg/mL. When tested against Gram-positive bacteria these agents exhibited an MIC of 2μg/mL (Table 2-4).

The most active chemical isolate was 3-O-acetoxy-3-epiisomasticadienolic acid with the MIC values ranged from 0.01 to 5μg/mL against all the strains of *H. pylori* and all other Gram-negative and Gram positive bacteria. This compound is found only in the acidic fraction of the kurdica gum (Table 1, 1.1 and 1.2). This compound is designated structure VIj (Table 6) and constitutes 9.00% of this fraction. Hypothetically, the higher activity of the acidic fraction of kurdica gum in comparison to acidic fractions of the other gums may be attributed to this compound (Sharifi, 2006). Antimicrobial activities of all the Structure IV, V and VI and their derivatives have not previously been reported (Table 6) thus the identification and characterisation of these compounds may represent an important finding that could lead to the development of a novel class of antimicrobial agents that may have application in the treatment of infectious disease.

Antibacterial agents can be bactericidal or bacteriostatic. The difference can be significant clinically. Bactericidal agents may be more effective in the treatment of disease particularly in immuncompromised individuals. The isolated components were all bactericidal (Ebrahimi *et-al*, 2008; Sharifi & Hazell, 2009).

The isolated components were divided into two major groups; Olean base skeleton, with three sub-group of Structure I, II and III (Table 5) and Lanosta Base skeleton with three sub-group of Structure IV, V and VI (Table 6). These components mimic steroid compounds, and the known antibiotic Fusidic acid. As a consequence an investigation of the possible mode of the action/s was undertaken which will be reported in a separate paper.

**Figure 2:**
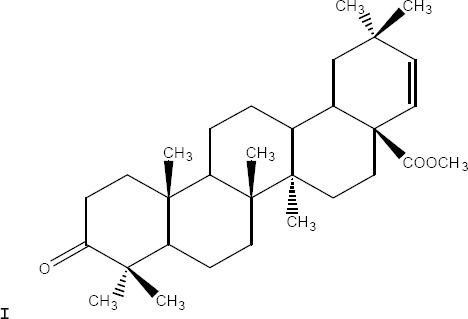
Methyl moronate

**Figure 3:**
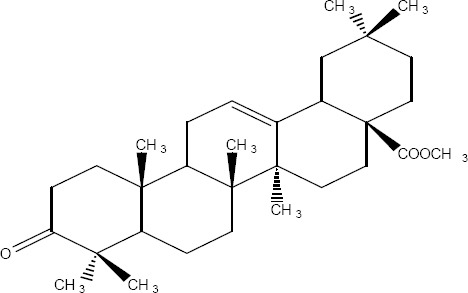
Methyl oleanonate

**Figure 4:**
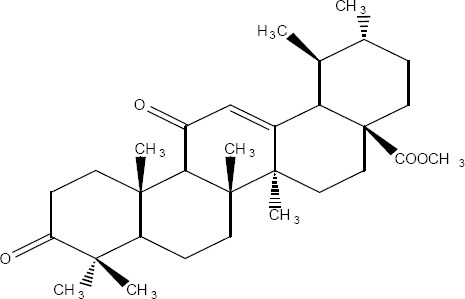
Ursonic acid (Methyl ester)

**Figure 5:**
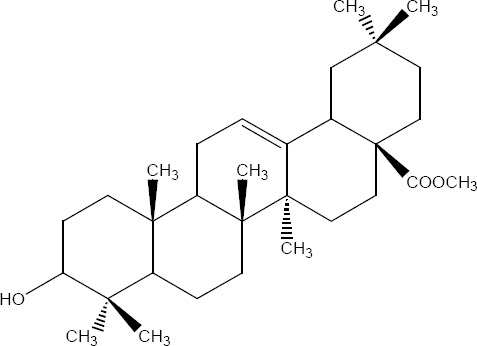
Methyl oleanolate

**Figure 6:**
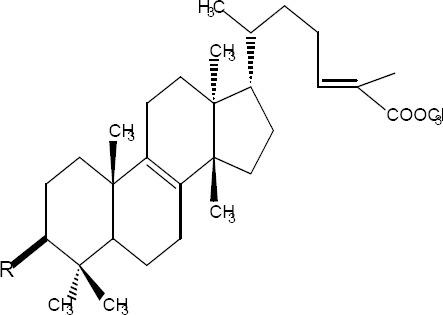
(R=O), Figure 9 (R=β-OH, H) and Figure 11 (α-CH3COO)

**Figure 7:**
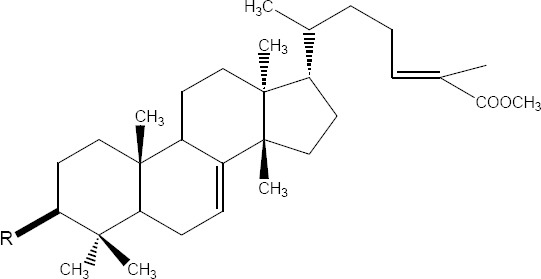
(R=O), Figure 8 (R=α-OH, H) and Figure 10 (α-CH3COO)
